# Improved Salt Tolerance in *Brassica napus* L. Overexpressing a Synthetic *Deinocuccus* Stress-Resistant Module DICW

**DOI:** 10.3390/ijms26062500

**Published:** 2025-03-11

**Authors:** Qilin Dai, Lingling Zhang, Shijie Jiang, Bodan Su, Zhaoqin Li, Yinying Shuai, Jin Wang

**Affiliations:** 1 College of Life Science and Agri-forestry, Southwest University of Science and Technology, Mianyang 621010, China; daiqilinmj@163.com (Q.D.); zll818427@163.com (L.Z.); lzq1356q@163.com (Z.L.); syy1232025@163.com (Y.S.); 2 Engineering Research Center of Biomass Materials, Ministry of Education, Southwest University of Science and Technology, Mianyang 621010, China; 3 National Key Laboratory of Agricultural Microbiology, Biotechnology Research Institute, Chinese Academy of Agricultural Sciences, Beijing 100081, China; subodan@caas.cn

**Keywords:** *Brassica napus* L., stress-resistant module DICW, salt stress, overexpression, physiology and biochemistry

## Abstract

Salt stress adversely impacts plant physiology by causing ionic, osmotic, and oxidative stress, ultimately hindering growth and yield. The genus *Deinococcus* contains unique stress resistance genes, and previous studies have shown that proteins such as IrrE, Csp, and WHy enhance stress tolerance in plants and microbial cells. However, their role in *Brassica napus* L. (oilseed rape) remains unexamined. In this study, a synthetic stress-resistance module, DICW, was constructed using the *Deinococcus*-derived genes *IrrE*, *Csp*, and *WHy* and heterologously overexpressed in *B. napus* to assess its impact on salt tolerance. The results demonstrated that the DICW module significantly improved seed germination and seedling growth under salt stress. Transgenic *B. napus* plants exhibited reduced membrane damage, higher leaf relative water content, enhanced accumulation of osmoregulatory substances, and elevated antioxidant enzyme activity compared to wild-type plants. Additionally, qRT-PCR analysis revealed the upregulation of stress-related genes (*BnRD29A*, *BnP5CS*, *BnKIN1*, *BnLEA1*, *BnNHX1*, and *BnSOS1*) and antioxidant enzyme-related genes (*BnSOD*, *BnPOD*, and *BnCAT*) in transgenic lines. In conclusion, the DICW module plays a crucial role in enhancing salt tolerance in *B*. *napus* by regulating stress responses and antioxidant mechanisms. This study provides valuable molecular insights into improving the survival and growth of *B. napus* in saline environments.

## 1. Introduction

Soil salinization is a major abiotic stress that severely limits crop yield and quality. Globally, approximately 954 million hectares (hm^2^) of agricultural land are affected by salinization, with an annual increase of 1–1.5 million hm^2^ [1,2]. Salt stress primarily impacts plants through osmotic stress and ionic toxicity. Osmotic stress arises from excessive Na^+^ concentrations in the soil, creating hypertonic conditions that hinder water and nutrient uptake, ultimately leading to plant dehydration [3]. Ionic toxicity occurs when salts absorbed by roots accumulate in plant tissues, disrupting ionic balance and interfering with metabolic processes [4]. These toxic effects dysregulate free radical metabolism in plant cells, leading to the accumulation of reactive oxygen species (ROS). High ROS concentrations cause oxidative damage to cell membranes, proteins, nucleic acids, and other cellular components, impairing cellular function and potentially resulting in cell death [5]. To mitigate the adverse effects of salt stress, plants have evolved complex defense mechanisms, including the increased accumulation of osmoregulatory substances, improved antioxidant enzyme activity, and the induced expression of stress-related genes. These mechanisms help maintain ionic balance and scavenge excessive ROS [6]. *Brassica napu* L. (oilseed rape) is the world’s second-largest oilseed crop, contributing 13–16% of the global vegetable oil production [7]. Despite its economic significance and widespread cultivation, soil salinity remains a major challenge to its productivity and profitability.

Advancements in synthetic biology have enabled the precise regulation of gene expression at the DNA, mRNA, and protein levels [8]. Researchers have also developed functional modules to enhance biological processes. For instance, integrating three functional modules into *E. coli* improved immunoglobulin production [9], while heterologous expression of the recombinant PCFF’O module in tobacco increased chrysoeriol production [10]. The overexpression of *SpSOS1* and *SpAHA1* in *Arabidopsis* conferred salt tolerance, with the co-expression of both genes leading to greater salt stress resistance compared to single-gene expression [11]. These findings underscore the potential of multi-gene co-expression in enhancing organism stress tolerance.

IrrE (PprI), a global regulatory protein from *Deinococcus radiodurans*, enhances tolerance to extreme environments by inducing *recA* and *pprA* gene expression and increasing peroxidase activity [12]. It also confers resistance to heat, radiation, oxidation, osmotic stress, and inhibitors in various organisms, including bacteria, fungi, plants, and mammalian cells [13]. Csp, found across a broad range of bacteria, function as RNA molecular chaperones that stabilize mRNA and facilitate translation under stress conditions such as nutrient deprivation and ionizing radiation [14]. WHy, a component of atypical late embryogenesis abundant proteins, accumulates under stress conditions such as drought, salt, extreme temperatures, and oxidative stress [15]. Its hydrophobic structural domain is linked to stress resistance in numerous organisms [16]. Studies have shown that transferring resistance genes from fungi or bacteria into plants can enhance abiotic stress tolerance [17]. For example, introducing *DgCspC* from *Deinococcus gobiensis* I-0 into cotton alleviated drought and salt stress damage [18], while the bacterial *popW* gene improved drought resistance in tobacco [19]. Similarly, transferring *IrrE* [20], *Csp* [21], and *WHy* [22] into plants has been shown to enhance salt stress tolerance. In this study, *IrrE*, *Csp*, and *WHy* were combined into a synthetic resistance module, DICW, using synthetic biology techniques. This module was integrated into the plant expression vector pBI121 and transformed into *Agrobacterium tumefaciens* EHA105. The DICW resistance system was then introduced into *B. napus* via *Agrobacterium*-mediated transformation to develop a new *B. napus* variety with enhanced salt tolerance.

## 2. Results

### 2.1. Generation of Transgenic B. napus Plants Overexpressing DICW

**Figure 1:**
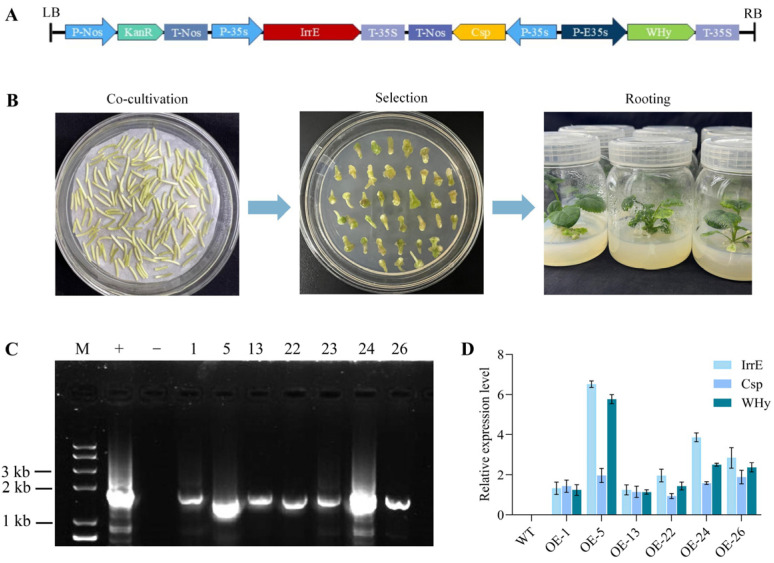
Molecular identification of the transgenic oilseed rape. (**A**) Schematic representation of the pBI121-DICW construct. (**B**) Transfection of DICW into oilseed rape. (**C**) PCR-based molecular identification of the transgenic oilseed rape. (**D**) Expression levels of DCIW in positive transgenic lines.

To investigate the function of DICW, a plant expression vector carrying DICW was constructed (Figure 1A), and trans-*DICW B. napus* plants were generated via the *Agrobacterium*-mediated transformation of oilseed rape hypocotyls (Figure 1B). A PCR analysis confirmed the presence of the transgene at the DNA level, identifying seven positive transgenic lines (Figure 1C). The expression levels of DICW in these lines were determined using qRT-PCR. The results showed that the wild-type oilseed rape exhibited no DICW expression, and was expressed in all the plants identified as positive (Figure 1D), from which we selected the three lines with high expression (OE-5, OE-24, and OE-26) for further study.

### 2.2. DICW Resistance-Module Promotes B. napus Growth Under Salt Stress

**Figure 2:**
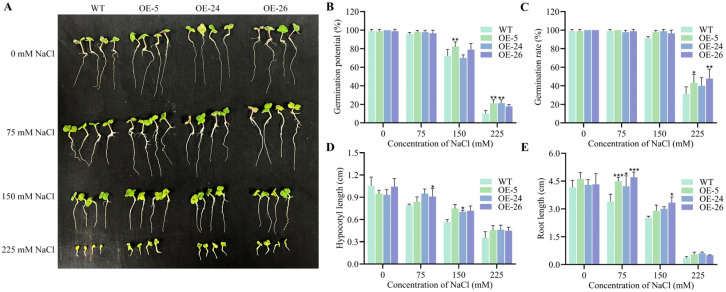
Salt stress tolerance analysis in the wild-type and *DICW* transgenic oilseed rape lines. (**A**) Phenotype comparison of the wild-type and transgenic lines after 7 d of exposure to different NaCl concentrations. (**B**) Germination potential after 3 d of treatment. (**C**) Germination percentage after 7 d of treatment. (**D**,**E**) Hypocotyl and root lengths after 7 d of treatment. The error bars indicate the standard deviation from three biological replicates (* *p* < 0.05; ** *p* < 0.01; *** *p* < 0.001).

To assess the impact of DICW on salt stress tolerance, we compared seed germination and seedling growth between the wild-type and *DICW* transgenic oilseed rape lines (OE-5, OE-24, and OE-26) under varying salt concentrations (0, 75, 150, and 225 mM NaCl). The results indicated no significant difference in growth between the transgenic and wild-type plants under normal conditions (Figure S1). Upon exposure to salt stress, both the wild-type and transgenic plants exhibited inhibited germination and growth, with the inhibition being more severe in the wild-type plants (Figure 2A). At 225 mM NaCl, both the germination potential and germination rate were significantly suppressed in the wild-type and transgenic plants, but the transgenic lines demonstrated higher germination potential and germination rates compared to wild-type plants (Figure 2B,C). Under 75 mM NaCl stress, the *DICW* transgenic seedlings exhibited significantly longer roots than the wild-type seedlings. At 250 mM NaCl, both hypocotyl length and root growth were completely inhibited in both the wild-type and transgenic plants (Figure 2D,E).

### 2.3. DICW Resistance Module Enhances Salt Tolerance in B. napus

**Figure 3:**
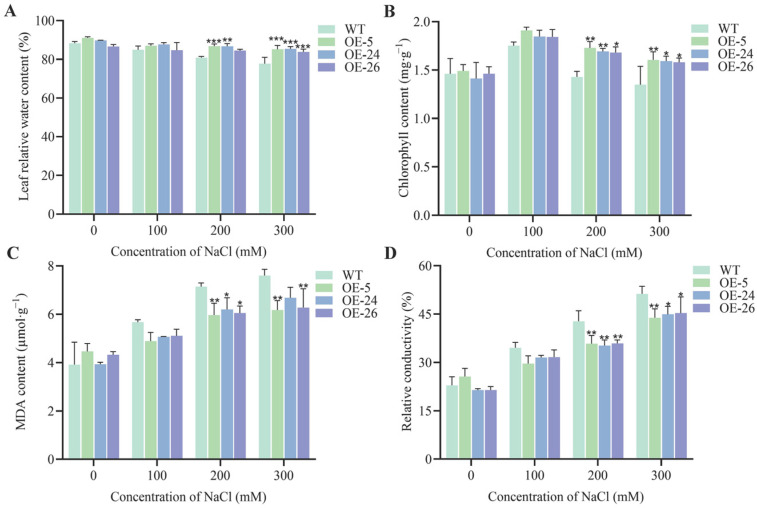
Enhanced salt tolerance in oilseed rape by the DICW resistance module. (**A**) Relative water content of the leaves under different NaCl concentrations. (**B**) Chlorophyll content of the leaves under different NaCl concentrations. (**C**) MDA content of the leaves under different NaCl concentrations. (**D**) Relative conductivity of the leaves under different NaCl concentrations. The error bars indicate standard deviations from three biological replicates (* *p* < 0.05; ** *p* < 0.01; *** *p* < 0.001).

When the transgenic and non-transgenic oilseed rape seedlings were subjected to stress treatments, no significant differences were observed in the leaf relative water content, total chlorophyll content, malondialdehyde (MDA) levels, or relative conductivity under normal conditions. However, under salt stress, the transgenic oilseed rape lines exhibited enhanced stress tolerance compared to the wild-type plants. Under 300 mM NaCl stress, the transgenic lines (DICW) maintained significantly higher leaf relative water content and total chlorophyll content than the wild-type plants (Figure 3A,B). The MDA levels and relative conductivity, indicators of membrane damage, increased in the wild-type and transgenic seedlings under salt stress but remained consistently lower in transgenic lines. Under 200 mM NaCl stress, the MDA levels in the transgenic seedlings were significantly lower than those in the wild-type plants, showing reductions of 16.51%, 13.23%, and 15.37% in the lines OE-5, OE-24, and OE-26, respectively. Similarly, relative conductivity decreased by 16.33%, 17.65%, and 16.1% in the same lines (Figure 3C,D).

**Figure 4:**
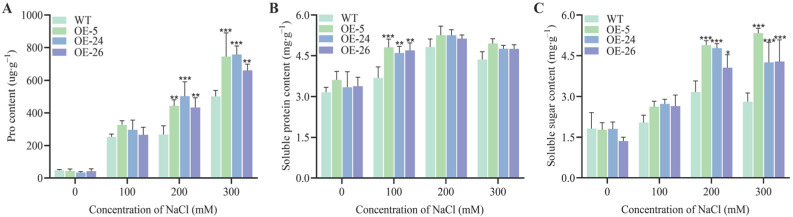
Osmoregulatory substance accumulation in the oilseed rape leaves under different NaCl stresses. (**A**) Pro content under different NaCl concentrations. (**B**) Soluble protein content under different NaCl concentrations. (**C**) Soluble sugar content under different NaCl concentrations. The error bars indicate standard deviations from three biological replicates (* *p* < 0.05; ** *p* < 0.01; *** *p* < 0.001).

In addition, osmoregulatory substances were measured across all the strains. Under non-stress conditions, no significant differences were detected between the wild-type and transgenic oilseed rape leaves. However, following salt stress exposure, the accumulation of osmoregulatory substances increased rapidly in both the wild-type and transgenic plants. After 200 mM NaCl stress, transgenic lines exhibited significantly higher proline (Pro) and soluble sugar contents than in wild-type plants (Figure 4A,C). Under 100 mM NaCl stress, the soluble protein content in the transgenic lines was significantly higher than in the wild-type plants, and with increasing concentration, the soluble protein of the *DICW* transgenic oilseed rape leaves was still higher than that of the wild-type plants (Figure 4B).

### 2.4. Antioxidant Capacity of B. napus Is Enhanced by the Resistance-Module DICW

**Figure 5:**
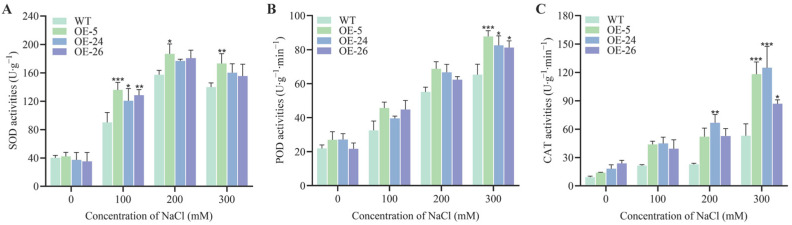
ROS scavenging capacity mediated by the DICW resistance module. (**A**) SOD activity in the oilseed rape under different NaCl conditions. (**B**) POD activity in the oilseed rape seedlings under different NaCl stresses. (**C**) CAT activity in the oilseed rape seedlings under different NaCl stresses. The error bars indicate the standard deviation of three biological replicates (* *p* < 0.05; ** *p* < 0.01; *** *p* < 0.001).

Reactive oxygen species (ROS) are generated when plants are exposed to abiotic stresses, leading to oxidative damage in plant cells. Antioxidant enzymes, such as superoxide dismutase (SOD), peroxidase (POD), and catalase (CAT), play a crucial role in scavenging ROS. To assess this response, we measured their enzyme activities. The results indicated a rapid increase in antioxidant enzyme activity in the plants subjected to salt stress. Under 100 mM NaCl stress, the SOD activity in the transgenic oilseed rape leaves was significantly higher than in the wild-type (Figure 5A). Under 300 mM NaCl stress, the POD and CAT activities in all the *DICW* transgenic oilseed rape lines were significantly elevated compared to the wild-type. Specifically, the POD enzyme activity in the *DICW* transgenic oilseed rape lines was higher by 34.49%, 26.38%, and 24.46%, respectively (Figure 5B), while the CAT enzyme activity increased by 122.8%, 135.97%, and 64.18%, respectively (Figure 5C).

### 2.5. Expression of Stress-Related Genes in DICW Transgenic Oilseed Rape Lines Under Salt Stress

**Figure 6:**
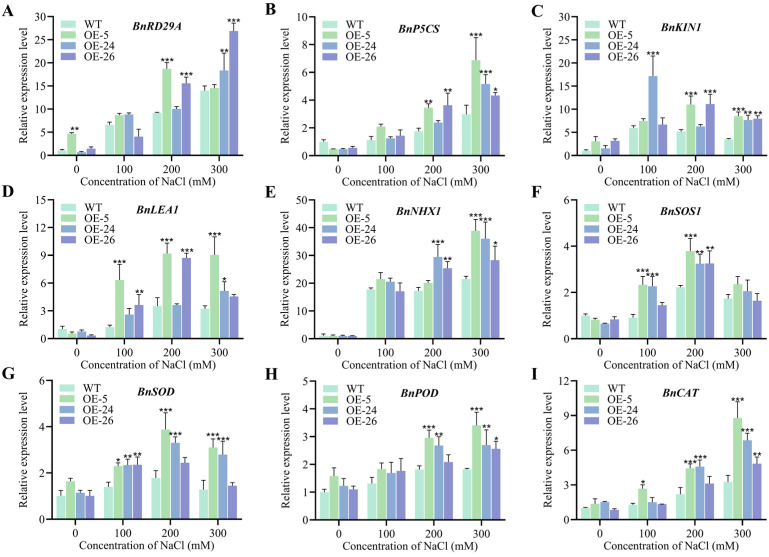
Expression analysis of stress-related genes in oilseed rape. (**A**–**I**) represent the expression levels of *BnRD29A*, *BnP5CS*, *BnKIN1*, *BnLEA1*, *BnNHX1*, *BnSOS1*, *BnSOD*, *BnPOD*, and *BnCAT*, respectively. The error bars indicate the standard deviation of three biological replicates (* *p* < 0.05; ** *p* < 0.01; *** *p* < 0.001).

To further explore the mechanisms by which DICW enhances salt tolerance in oilseed rape, we analyzed the expression patterns of several genes under normal and salt stress conditions (Figure 6). We examined the genes associated with stress response (*BnRD29A*, *BnP5CS*, *BnKIN1*, *BnLEA1*, *BnNHX1*, and *BnSOS1*) and the genes related to antioxidant enzymes (*BnSOD*, *BnPOD*, and *BnCAT*). Under normal conditions, the DICW resistance module had no significant effect on these genes in *B. napus* However, upon exposure to salt stress, the expression of all the antioxidant genes was upregulated. The expression of *BnP5CS*, *BnKIN1*, *BnNHX1*, *BnPOD*, and *BnCAT* was higher in the *DICW* transgenic oilseed rape lines compared to the wild-type, especially under high salt stress.

## 3. Discussion

Soil salinization is a significant agricultural problem, as high levels of salt stress can severely impact plant growth [23]. Traditional breeding methods have proven ineffective in enhancing plant stress tolerance, leading to the adoption of transgenic techniques to improve abiotic stress tolerance in economically important crops [24]. Research highlights the role of specific proteins IrrE, Csp, and WHy in increasing resistance to adverse conditions. IrrE is known for regulating DNA damage repair [13], while Csp is a multifunctional RNA-binding protein [25]. WHy is a component of the DrwH protein, with its gene expression upregulated under oxidative and salt stress conditions by IrrE [15]. In this study, these proteins were biosynthesized to form a resistance module called DICW, which aims to enhance organism resistance by functioning through the DNA–RNA–protein pathway. Therefore, the DICW module was transferred into *B. napus* for heterologous expression, and its function was evaluated by assessing the salt tolerance of the transgenic oilseed rape plants. Based on seed germination, seedling growth phenotype, physiology, biochemistry, and stress gene expression in oilseed rape, we analyzed the role of DICW in salt tolerance in *B. napus*.

Salt stress negatively affects seed germination and root growth, which are essential for water uptake and overall plant development [26,27]. In the study, the transgenic oilseed rape lines expressing DICW exhibited higher germination rates and longer root lengths under salt stress compared to the wild-type plants, suggesting that DICW mitigates the inhibitory effects of salt stress (Figure 2). Therefore, DICW promotes seed germination in *B. napus* under salt stress and alleviates its inhibitory impact on root growth. The co-expression of five genes including *NCED3*, *ABAR*, *CBF3*, *LOS5*, and *ICE1* in *B. napus* significantly enhanced seed germination and growth under drought, salt, and low-temperature stresses [28], indicating their role in stress tolerance. Similarly, the co-expression of the *AVP1/PP2A*-*C5/AtCLCc* plants in *A. thaliana* resulted in improved seed activity and growth under salt and drought stresses compared to the wild-type plants [29]. These findings suggest that the expression of multiple genes can synergistically enhance a plant’s ability to withstand abiotic stresses. This multi-gene approach may be more effective than single-gene modifications, as it targets multiple pathways involved in stress responses.

This study also examined the effects of high salt stress (high Na^+^ concentration in soil) on plants, focusing on the physiological responses of transgenic oilseed rape lines (DICW) compared to wild-type plants. High Na^+^ levels reduce water availability around plant roots, leading to water stress [30]. Additionally, salt stress disrupts chlorophyll synthesis by affecting enzyme activity, resulting in lower chlorophyll content and reduced photosynthesis [31]. In this study, the transgenic oilseed rape lines (DICW) exhibited higher relative water content and chlorophyll levels under salt stress compared to the wild-type plants, indicating improved salt tolerance (Figure 3). Plants accumulate osmoregulatory substances like Pro, sugars, and proteins to mitigate the effects of abiotic stress [32]. Pro is one of the most abundant endogenous osmoregulators under salinity stress [33], acting as a metal chelator, antioxidant, osmotic agent, and signaling molecule, with higher levels often correlating with greater stress resistance [34]. Additionally, sugars and proteins play crucial roles in regulating cell division, preventing water loss, protecting chlorophyll, scavenging free radicals, and stabilizing membranes and proteins [35]. In this study, the DICW transgenic lines accumulated significantly higher levels of Pro and soluble sugars under salt stress compared to the wild-type plants. This suggests that the enhanced salt tolerance in the DICW lines may be due to altered physiological responses, including the increased accumulation of osmoregulatory substances (Figure 4).

When plants experience abiotic stress (e.g., salt, drought, or extreme temperatures), ROS levels increase rapidly, leading to detrimental effects such as protein denaturation, DNA mutations, and membrane lipid peroxidation, which damage plant cells [36]. SOD, POD, and CAT are key enzymes for scavenging ROS and protecting plant cells from oxidative damage [37]. MDA is a byproduct of membrane lipid peroxidation and serves as an indicator of oxidative damage. The extent of membrane damage in cells can often be assessed by measuring ion leakage [38]. High levels of MDA and higher conductivity suggest significant membrane damage. Numerous studies indicate that plants with higher SOD, POD, and CAT activity and lower MDA levels and relative conductivity exhibit greater stress tolerance. These traits help maintain cellular integrity and function under adverse conditions [39,40,41]. Our results showed that the DICW transgenic oilseed rape lines exhibited higher antioxidant enzyme activities (SOD, POD, and CAT) and lower MDA content and relative conductivity compared to the wild-type plants (Figure 3 and Figure 5). This suggests that DICW enhances the plant’s ability to cope with oxidative stress, possibly by regulating genes involved in the ROS signaling pathway and ion transport mechanisms. The regulatory effects of DICW help maintain ionic homeostasis under salt stress, contributing to improved salt tolerance in *Brassica napus*.

Stress-related genes (e.g., BnRD29A, *BnP5CS*, *BnKIN1*, and *BnLEA1*) play crucial roles in plant responses to salt stress and are significantly upregulated under such conditions [42,43,44,45,46], which is consistent with our experimental findings. Notably, the DICW strain exhibited higher expression levels of these genes compared to the wild-type, indicating that DICW positively regulates their expression. This suggests that DICW enhances a plant’s ability to cope with salt stress by activating stress-responsive pathways. We also examined the expression of salt stress-related genes such as *BnNHX1* and *BnSOS1* [47] and antioxidant enzyme-related genes such as *BnSOD*, *BnPOD*, and *BnCAT*. *BnNHX1* (vesicular membrane Na^+^/H^+^ antiporter) and *BnSOS1* (plasma membrane Na^+^/H^+^ transporter) are critical for maintaining ion homeostasis by regulating Na^+^ transport under salt stress [48]. The expression of *BnNHX1* and *BnSOS1* is significantly induced upon exposure to salt stress [49]. In this study, the upregulation of these genes in the DICW strain suggests that DICW enhances ion toxicity management, a key issue under high salinity. The findings align with previous studies in other crops (e.g., tomato, rice, soybean, and maize), where high expression of stress-related and antioxidant genes correlates with improved stress tolerance. This suggests that the mechanisms observed in oilseed rape may be conserved across species and that DICW-like strategies could be applied to improve salt tolerance in other crops. It is well established that under abiotic stress, plants enhance their ability to scavenge ROS by increasing the activity of antioxidant enzymes. This process is closely linked to the stress-induced expression of antioxidant enzyme genes (*BnSOD*, *BnPOD*, and *BnCAT*) [50]. These genes encode antioxidant enzymes (superoxide dismutase, peroxidase, and catalase) that scavenge reactive oxygen species (ROS), which accumulate under abiotic stress and cause cellular damage. The findings are consistent with previous studies in other crops (e.g., tomato, rice, soybean, and maize), where elevated expression of stress-related and antioxidant genes correlates with enhanced stress tolerance [51,52,53,54]. The DICW strain exhibited significant upregulation of these genes under salt stress, suggesting that DICW enhances the plant’s ROS-scavenging capacity, thereby reducing oxidative damage. Under normal conditions, the gene expression levels in DICW oilseed rape remained comparable to those in wild-type plants, indicating that DICW’s regulatory effects are specifically induced by stress. The significant upregulation of these genes under salt stress highlights DICW’s role in activating stress-responsive mechanisms only when needed, which could be an energy-efficient strategy for stress adaptation.

The remarkable potential of the DICW resistance module in improving salt tolerance in plants provides a scientific foundation for the development and promotion of transgenic crops in saline environments. The introduction of transgenic crops carrying the DICW module in such areas may improve crop yields and quality, thereby increasing farmers’ incomes and improving food security. However, the potential ecological consequences must be carefully evaluated. Crops with enhanced salt tolerance could exert competitive pressure on native plant communities or even become invasive, thereby posing a threat to biodiversity [55]. Therefore, while the implementation of the DICW resistance module in saline soils is expected to enhance salt resilience, its potential economic and ecological impacts require comprehensive assessment through long-term monitoring and in-depth studies.

## 4. Materials and Methods

### 4.1. Plant Material and Growing Conditions

Wild-type *B. napus* seeds (84100-18) were obtained from the Genetics Laboratory of Sichuan University. All the oilseed rape seedlings were cultivated in a tissue culture room at 25 °C under a 16 h light/8 h dark photoperiod. The expression vector pBI121 and *Agrobacterium tumefaciens* EHA105 were maintained in our laboratory.

### 4.2. Construction and Transformation of Expression Vectors

The complete sequence of the *DICW* nucleic acid was synthesized through artificial chemical methods. The recombinant plasmid pJET-DICW, designed with an anti-stress function, was constructed. The anti-stress module DICW fragment was extracted via double digestion using *Eco*R I and *Hin*d III. The fragment was then ligated into the pBI121 vector to generate the plant expression vector pBI-DICW, which was introduced into *Agrobacterium tumefaciens* EHA105. Transformation into *B. napus* was performed using *Agrobacterium*-mediated methods.

### 4.3. Positive Identification of Transgenic Plants

The initial identification of transgenic plants was performed using PCR analysis followed by qRT-PCR to determine DICW expression levels. The three lines exhibiting the highest expression levels were selected for further experimentation.

### 4.4. Determination of Seed Germination Index

Seeds of the wild-type and *DICW* transgenic oilseed rape lines were surface-sterilized and placed on Petri dishes lined with two sterile filter papers, with 30 seeds per dish. The seeds were treated with 5 mL of NaCl solutions at concentrations of 0, 75, 150, and 225 mM. Each treatment was repeated three times. The dishes were incubated at 25 °C under a 16 h photoperiod. Germination was defined as the emergence of an embryonic root of at least 2 mm. Germination potential (GP) and germination rate (GR) were assessed on days 3 and 7, respectively. Radicle and embryo axis lengths were measured on day 7. GP and GR were calculated as follows:

$$\mathrm{GP}\;(\%)=\frac{\mathrm{Number}\;\mathrm{of}\;\mathrm{germinated}\;\mathrm{seeds}\;\mathrm{on}\;\mathrm{day}\;3}{\mathrm{Total}\;\mathrm{number}\;\mathrm{of}\;\mathrm{seeds}\;\mathrm{in}\;\mathrm{the}\;Petri\;\mathrm{dish}}\;\times 100$$

$$\mathrm{GR}\;(\%)=\frac{\mathrm{Number}\;\mathrm{of}\;\mathrm{germinated}\;\mathrm{seeds}\;\mathrm{on}\;\mathrm{day}\;7}{\mathrm{Total}\;\mathrm{number}\;\mathrm{of}\;\mathrm{seeds}\;\mathrm{in}\;\mathrm{the}\;Petri\;\mathrm{dish}}\times 100$$

### 4.5. Measurement of Physiological Indicators

Following sterilization, both the wild-type and transgenic seeds were uniformly sown on the Murashige and Skoog (MS) medium. After observing germination and root development, uniform seedlings were selected and transplanted into 9.3 × 7.5 cm pots containing a mixture of field soil and nutrient soil (2:1, *w*/*w*). Each pot containing three seedlings was irrigated with MS nutrient solution every three days. After 30 days, salt stress treatments were applied. Both wild-type and transgenic plants were subjected to NaCl concentrations of 0, 100, 200, and 300 mM. Each treatment received 100 mL of the respective saline solution, with plastic sheeting placed at the bottom to prevent leakage. After 48 h, leaf samples were collected for physiological and biochemical analyses. Each experiment was repeated three times.

The relative water content of the leaves was measured following the method described by Patanè [56]. The total chlorophyll content was assessed according to Gerona’s protocol [57]. Malondialdehyde, relative conductivity, proline, soluble sugars, and soluble proteins were quantified using the methods of Zhu [58]. The activities of antioxidant enzymes, including SOD, POD, and CAT, were determined following the procedures of Li [59].

### 4.6. qRT-PCR Analysis of Stress-Related Genes

Total RNA was extracted from *B. napus* and reverse-transcribed into cDNA. The expression levels of stress-related genes were assessed using qRT-PCR. The β-Actin gene was used as an internal control. The relative expression levels were calculated using the 2^−ΔΔCt^ (Livak method). Each sample underwent three biological replicates. The primers used in this study are listed in Supplementary Table S1.

### 4.7. Statistical Analyses

The data were statistically analyzed and visualized using Microsoft Excel 2019 (Microsoft Corp, Seattle, WA, USA) and the GraphPad Prism 10 (GraphPad Software, San Diego, CA, USA) software. All the experiments were repeated at least three times. The results are presented as mean ± standard deviation. Student’s *t*-test was used; * *p* < 0.05, ** *p* < 0.01, and *** *p* < 0.001 for significant differences.

## 5. Conclusions

Our findings demonstrate that the DICW stress tolerance module promotes seed germination and seedling growth, mitigates membrane damage, and enhances the accumulation of osmoregulatory substances and antioxidant enzyme activity under salt stress conditions. The qRT-PCR analysis revealed that DICW upregulated the expression of the salt stress-related gene (*BnRD29A*, *BnP5CS*, *BnKIN1*, *BnLEA1*, *BnNHX1*, and *BnSOS1*) and antioxidant enzyme gene (*BnSOD*, *BnPOD*, and *BnCAT*) expression. Collectively, these results indicate that DICW significantly enhances salt tolerance in *B. napus* by modulating the expression of multiple salt tolerance-related genes and their physiological mechanisms.

## Data Availability

The original data presented in the study are included in the article and Supplementary Materials; further inquiry can be addressed to the corresponding author.

## Supplementary Materials

The following supporting information can be downloaded at https://www.mdpi.com/article/10.3390/ijms26062500/s1.
