# Supplementary material for: Improved Salt Tolerance in Brassica napus L. Overexpressing a Synthetic Deinocuccus Stress-Resistant Module DICW
Source: Int J Mol Sci. 2025 Mar 11;26(6):2500. doi: 10.3390/ijms26062500 (PMC11942316; doi:10.3390/ijms26062500)
Supplement: Supplementary file 1 [file ijms-26-02500-s001.zip › ijms-3445019-supplementary.pdf]

Supplementary Materials

Improved Salt Tolerance in *Brassica napus* L. Overexpressing a Synthetic *Deinocuccus* Stress-Resistant Module DICW

Qilin Dai [1,2,†], Lingling Zhang [1,2,†], Shijie Jiang [1,2,*], Bodan Su [3], Zhaoqin Li [1,2], Yinying Shuai [1,2] and Jin Wang [3,*]

[1] College of Life Science and Agri-forestry, Southwest University of Science and Technology, Mianyang 621010, China; daiqilinmj@163.com (Q.D.); zll818427@163.com (L.Z.); lzq1356q@163.com (Z.L.); syy1232025@163.com (Y.S.)
[2] Engineering Research Center of Biomass Materials, Ministry of Education, Southwest University of Science and Technology, Mianyang 621010, China
[3] National Key Laboratory of Agricultural Microbiology, Biotechnology Research Institute, Chinese Academy of Agricultural Sciences, Beijing 100081, China; subodan@caas.cn
* Correspondence: sjjiang0406@swust.edu.cn (S.J.); wangjin@caas.cn (J.W.)
† These authors contributed equally to this work.

Table S1. List of primers used in this study

Primer name	Primer sequence (5'-3')	Application
DICW-F	GCCGCCCAGTGATTTCTCCATAA	Gene cloning
DICW-R	CATCGCCATTCAGCGTGTCCTCT	Gene cloning
IrrE-F	CCGGGCGTGGACCTGAAGTT	qRT-PCR
IrrE-R	CCTCGTAGGCGTCGTGGATGT	qRT-PCR
Csp-F	GTGAAGTGGTTCAACGCCGA	qRT-PCR
Csp-R	TCTTGAAGCCGCTGCCCT	qRT-PCR
WHy-F	CACAGCGGAGCCAAGCAGAG	qRT-PCR
WHy-R	TGGTGGCGGTCTTGAAGGTC	qRT-PCR
BnRD29A-F	ATTATTGGCTCGGTGGTA	qRT-PCR
BnRD29A-R	GCTAAGTGGTTGTGATGAC	qRT-PCR
Bnp5CS-F	CAGAAGCCACAGACTGAACTTG	qRT-PCR
Bnp5CS-R	AAACTGCTATCAGTCACCAGCA	qRT-PCR
BnKIN1-F	ATATGCTGCTCCTGAATA	qRT-PCR
BnKIN1-R	TCTTCCTGATATTAGTTCCA	qRT-PCR
BnLEA1-F	GTGGTGCCGTATAGTATA	qRT-PCR
BnLEA1-R	ACAACAGGAAGATCAATG	qRT-PCR
BnNHX1-F	CCTTGCTTGGTGTTGCTA	qRT-PCR
BnNHX1-R	TCGGTCAGTTGAGTGTCT	qRT-PCR
BnSOS1-F	AAGGGGAGTCGCTGATGAATG	qRT-PCR
BnSOS1-R	GAGCCAAAGAACCGAGACAATG	qRT-PCR
BnSOD-F	GTTCAACGGCGGAGGTCA	qRT-PCR
BnSOD-R	AAGGGGAGTCGCTGATGAATG	qRT-PCR
BnPOD-F	GGCATGTATTATGTTTCGTGCGTCTC	qRT-PCR
BnPOD-R	GCGTCACAACCATTGACAAAGCAG	qRT-PCR
BnCAT-F	GAAGGTTTCGGCGTCCACA	qRT-PCR
BnCAT-R	TTGGTCACATCAAGCGGGTC	qRT-PCR
β-actin-F	ACTGTGCCAATCTACGAGGGTT	Reference gene
β-actin-R	TCTTACAATTTCCCGCTCTGCT	Reference gene

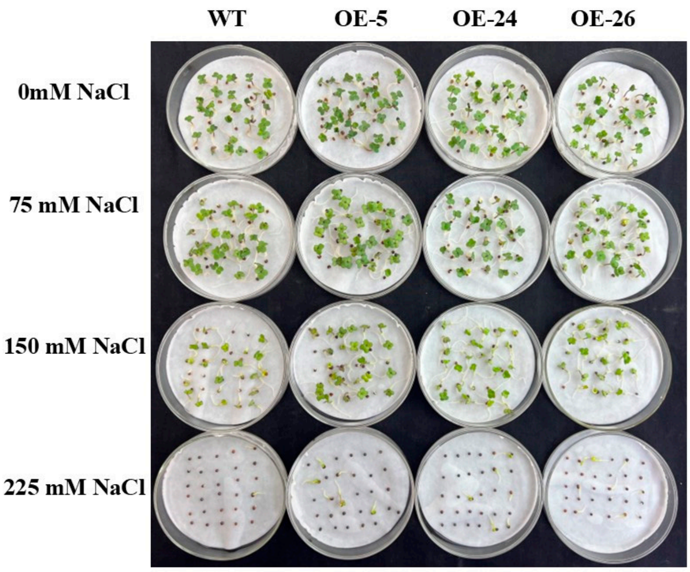

Figure S1. Germination phenotype of WT and DICW-OE seeds under different salt stress conditions.
